# Application of Water Treated with Low-Temperature Low-Pressure Glow Plasma for Quality Improvement of Barley and Malt

**DOI:** 10.3390/biom10020267

**Published:** 2020-02-10

**Authors:** Aneta Pater, Marek Zdaniewicz, Paweł Satora, Gohar Khachatryan, Zdzisław Oszczęda

**Affiliations:** 1 Department of Fermentation Technology and Microbiology, Faculty of Food Technology, University of Agriculture, Balicka Street 122, 30-149 Kraków, Poland; m.zdaniewicz@ur.krakow.pl (M.Z.); pawel.satora@urk.edu.pl (P.S.); 2 Department of Food Quality Analysis and Evaluation, Faculty of Food Technology, University of Agriculture, Balicka Street 122, 30-149 Kraków, Poland; rrgchacz@cyf-kr.edu.pl; 3 Nantes Nanotechnological Systems, Dolne Młyny Street 21, 59-700 Bolesławiec, Poland; oszczeda@nantes.com.pl

**Keywords:** plasma-treated waters, barley, energy and germination capacity, water uptake capacity of the grain, malting

## Abstract

The aim of this study is to determine the quality of water treated with low-temperature, low-pressure glow plasma, either in the air or under nitrogen, in order to obtain high-quality brewer’s malt. To this end, plasma-treated spring water was used for barley grain soaking. In two-row spring barley grain, the procedure provided significantly higher water uptake capacity and grain sensitivity to water, as well as energy and germination capacity. The resulting malt showed improved moisture and 1000-grain mass. Furthermore, laboratory wort produced from the malt by the congress method did not differ statistically from a control sample in terms of filtration time, pH, turbidity, color, extract, free amino nitrogen compounds, and aromatic composition.

## 1. Introduction

Positive results in grain germination research [1] have led to the analysis of its applicability in brewing malt production processes, where germination plays a key role. Barley malt is the most important and necessary raw material in beer production. Generally, it is produced from two-row spring barley. Growing consumer demand for new beers has brought more attention to malts produced from other cereals, such as oat, wheat, buckwheat, and rye. However, barley malt has been historically accepted as the most common and appropriate cereal for the production of beer wort [2,3]. Due to the absence of active enzymes necessary for the hydrolysis of extractive components, barley seeds produce an amino-acid-poor extract of very high viscosity, which is also poor in adequate flavor and taste quality that is typical for beer [4]. Therefore, conducting the germination process in appropriate conditions is essential for the above-mentioned parameters, changing the biological, physical, and chemical properties of the seed and providing appropriate laxity [5]. The quality criteria for barley seed selected for brewing malt production are very precise. The brewing industry has imposed requirements and expects malthouses to deliver materials with high stability of basic seed components and performance characteristics [6]. The maximum protein concentration in barley used for the brewing malt production reaches 11.5%. In some cases, this upper limit is difficult to maintain, due to varying protein content in the seed. Additionally, the crop-growing method, environment, and weather conditions often influence the protein content in brewing barley and are thus critical to consider [7].

Appropriately prepared malt, characterized by high-quality parameters, guarantees the proper progress of the technological process (i.e., short mashing time, the optimum composition of wort and beer, and highly efficient production) [8]. Germinated seed represents a natural source of amylolytic enzymes, such as alpha-amylases (solubilizing and dextrinizing enzymes) and beta-amylases (saccharifying enzymes) [9]. The above-mentioned enzymes are required in starch hydrolysis during the mashing process. They determine the selection of sugars (e.g., maltose) that may be used by the yeast during the fermentation process. The quality of barley for brewing is determined in lab malthouses. Extractivity—the level of extract obtained from the process, also known as extract efficiency—is a basic malt quality factor [10]. 

An instrument has recently been constructed to produce low-temperature, low-pressure glow plasma at low frequency (LPGP) [11], which was first used for water treatment. Białopiotrowicz et al. [12] reported the structure and selected physicochemical properties of water exposed to LPGP in the air. Studies have shown that water treated with LPGP changed its physical and selected physiochemical properties. That water contained aqueous clathrates with incorporated singlet oxygen molecules. For their size, these clathrates more readily permeated cell membranes, transporting excited oxygen molecules into the cells and tissues. These studies were then extended to the treatment of water with LPGP under nitrogen [13]. The stimulation of pathogenicity and reproduction of entomopathogenic fungi with such water was demonstrated. The positive effects of water treated with LPGP on soil micro-organisms and, hence, on the growth of various plants, providing enhanced crops and their quality, was described in a monograph by Tomasik [14]. Watering herbs with water treated with LPGP prepared in the air considerably changed the composition of the essential oils produced by peppermint [15]. Subsequently [16], treating water with LPGP under oxygen-free nitrogen was carried out. Depending on the time of the treatment, such water formed aqueous clathrates incorporating molecular nitrogen in its various excited states. Interesting effects of water treated with LPGP under nitrogen on the yield and composition of essential oils from basil have also been observed [17]. 

Such results of LPGP-treated water prompted us to check its applicability in producing high-quality brewer’s malt. Particular attention is paid to the processes occurring in the malthouse and their impact on subsequent processes in the brewhouse (i.e., of a brewery). 

It should be mentioned that various plasmas are available depending on the ways of their generation. Hence, there are different areas of their potential application. Recently reported plasmas [18,19,20,21,22,23,24] resemble LPGP neither in the methods of their generation nor in their character. 

## 2. Materials and Methods 

### 2.1. Materials 

A concerto two-row barley variety with 11.75% moisture content and commercially available Żywiec Zdrój spring water were used.

### 2.2. Methods 

#### 2.2.1. Water Treated with LPGP in the Air 

A total of 1500 mL of spring water (Żywiec Zdrój) in 2000-mL open Pyrex glass bottles was placed in a reactor chamber closely to a lamp generating plasma and exposed to plasma for 30 min. Plasma at 38 °C was generated in the lamp at 5 × 10^−3^ mbar, 600 V, 50 mA, and 10 kHz frequency. The chamber with the water sample remained at normal pressure. The water produced was stored at ambient temperature in 2000-mL closed Teflon containers. 

#### 2.2.2. Water Treated with LPGP under Nitrogen 

**Figure 1:**
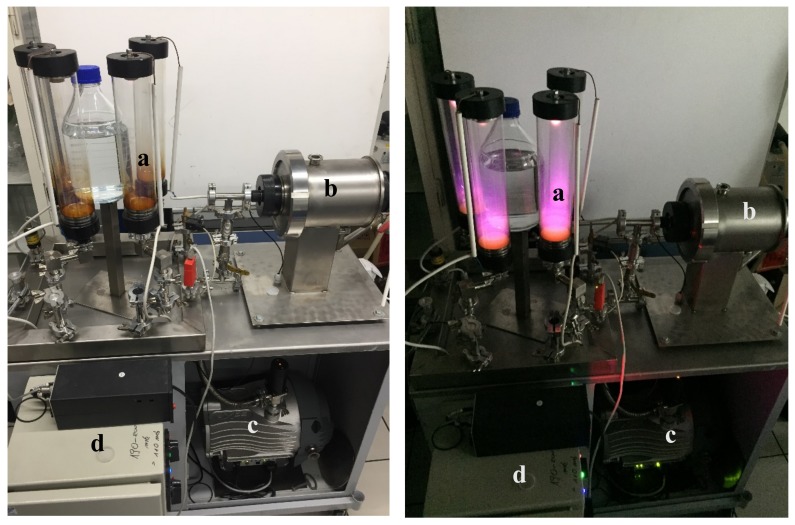
Plasmothrone (without cover) used in this study. (**a**) Lamps, (**b**) pulse generator, (**c**) vacuum pump, (**d**) power supply.

A total of 1500 mL of spring water (Żywiec Zdrój) was placed in 2000-mL open Pyrex glass bottles and a stream of nitrogen was bubbled through it for 15 min. The nitrogen was deoxygenated by passing it through an absorber filled with an alkaline solution of resorcinol. After placing the bottles in the reactor, its chamber and the free space over the liquid were additionally filled with deoxygenated nitrogen. The conditions of treatment with LPGP and the storage of the product are the same as described in Section 2.2.1. The plasmothrone used in this study is visualized in Figure 1.

#### 2.2.3. Barley Malt 

**Figure 2:**
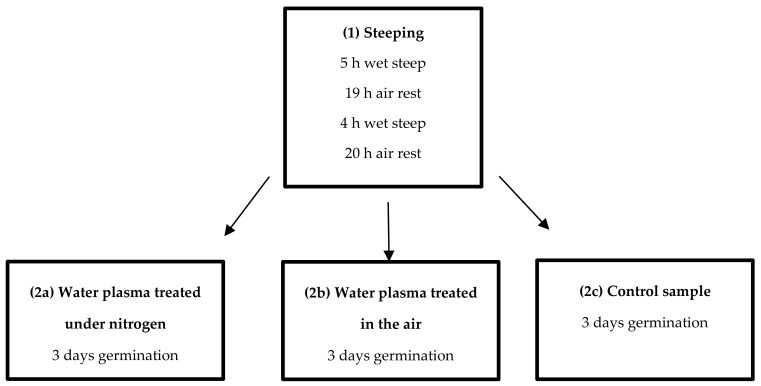

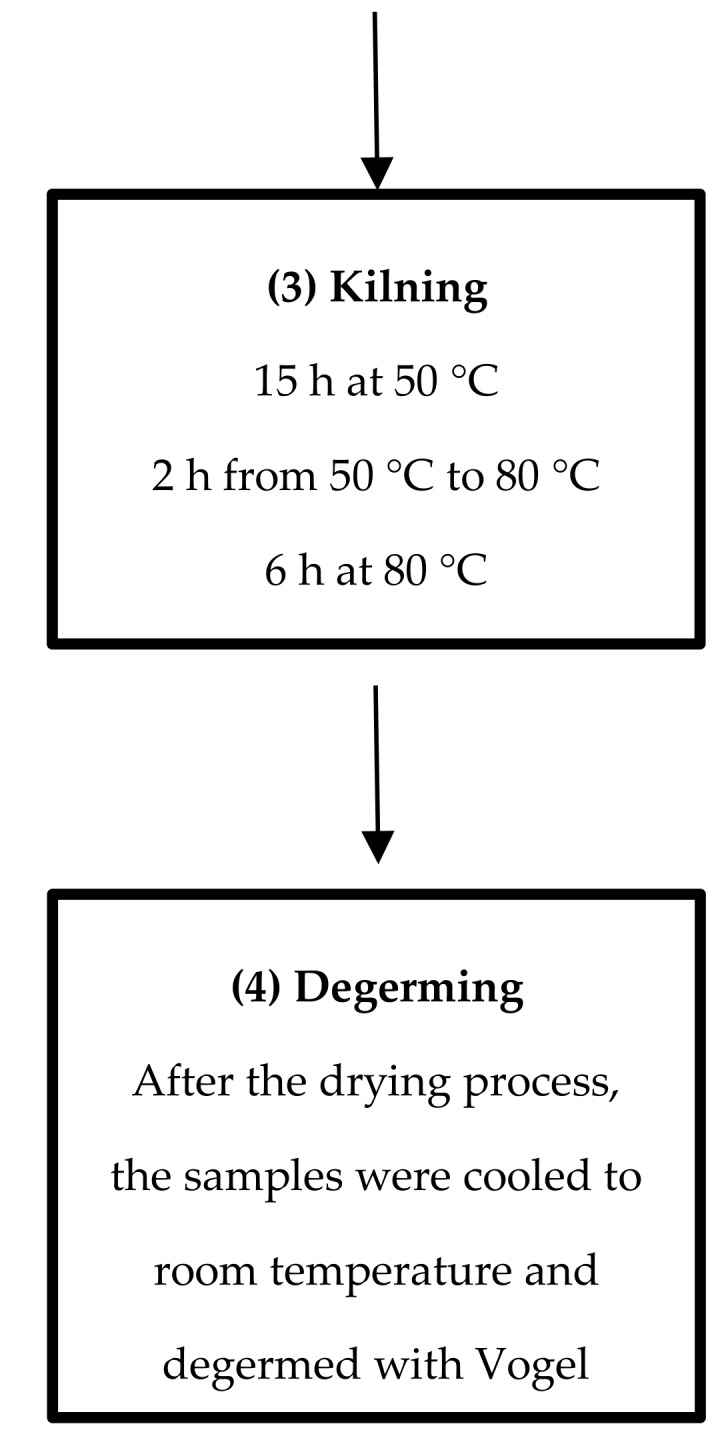
Malting process scheme.

Before the analysis, two-row spring barley variety seeds were sorted and purified. The malting process of the prepared barley involved water treated with LPGP, as described in Section 2.2.1 and Section 2.2.2. A Pilsner-type malt was obtained as a result of the 6-day malting of 200 g brewing barley seed (Figure 2) in lab conditions at the Department of Fermentation Technology and Microbiology of the University of Agriculture in Kraków. The malting process proceeded in five stages, according to the instructions prepared by the Experimental Station for the Evaluation of Varieties (the Chem and Tech Lab) in Słupia Wielka, Poland [25]. 

#### 2.2.4. FTIR Spectral Characteristics of LPGP-Treated Waters 

The FTIR–ATR spectra of samples were recorded in the range of 4000–700 cm^−1^ at a resolution of 4 cm^−1^, using a Mattson 3000 FT–IR (Madison, WI, USA) spectrophotometer equipped with a 30SPEC 30° reflectance adapter fitted with the MIRacle ATR accessory from PIKE Technologies Inc., Madison, Wisconsin, USA. 

#### 2.2.5. Barley Analysis 

The water uptake capacity of the grain (W2) was tested by placing 200 g of sample barley (11.75% moisture) in a pot and soaking in 200 mL plasma-treated water. After predefined soaking times (5, 12, and 50 h), the sample was weighed (after draining). Seed soaking was calculated from the obtained masses using the following formula:

W2 = 100 − M1 ⋅ (100 − W1)/M2(1)

where M1 and M2 are the sample masses (kg) prior to and after soaking, respectively, and W1 and W2 are the original and after-soaking water contents (%), respectively. 

Schönfeld’s method was applied to determine the barley germination energy. Six portions of barley (500 seeds each) were placed in funnels and soaked in LPGP prepared either under nitrogen or in the air, or in non-treated water as a control. The funnels were covered with wet paper filters and Petri dishes. After 3 h, the water was drained and the seeds were left for 18 to 20 h. Then, the seeds were re-soaked in clamped funnels for 2 h. After 72 h, the non-germinated seeds were counted and soaked again in funnels for 30 min. The germination procedure was conducted for 120 h. Finally, the number of non-germinated seeds was determined [26]. 

Seed sensitivity to water was determined by placing an appropriate portion of barley (100 seeds) in Petri dishes, followed by soaking the grain in the analyzed waters (4 and 8 mL). The number of germinated seeds was determined after 48 h. 

#### 2.2.6. Malt Analysis 

Acrospire length was determined by placing 20 malt seeds in conical flasks containing 40 mL 2% CuSO_4_. The flasks were covered and boiled on a hot heating panel for 30 min. Subsequently, the samples were cooled, placed in Petri dishes, and the length of their acrospires was estimated. 

Seed moisture was analyzed by grinding malt in a malt graining lab mill for 12 s. Then, 4 g of the malt from the sample was taken and placed in a MAC 50 moisture analyzer [27]. 

The mass of 1000 malt seeds was determined by weighing two approximately 40 g portions of barley. After removing damaged seeds and foreign bodies, the number of seeds was estimated [28]. 

Grading by grain size was analyzed by placing 100 g of seed sample on the frame of a sorting machine with appropriately located sieves (2.2, 2.5, and 2.8 mm). The sorting machine was run for 5 min and, afterwards, useful and useless impurities were removed from each sieve and transferred onto the bottom cover. The purified seed fraction and the residue on the bottom were weighted separately [29]. 

Extractivity was measured by preparing Congress mash using the EBC lab method. The extract was determined in the wort and the extractivity of the resultant malt was calculated [30]. 

Determination of protein content was carried out using the Dumas method. Approximately 0.5 g of the analyzed material was placed on tin foil. The foil was sealed and placed in a charging head. Subsequently, the system was sealed and degassed. The sample was burnt at 950 °C in pure oxygen. The combustion products were directed to a secondary afterburner (850 °C) for final oxidation and removal of impurities. Gases from the combustion process were stored inside a ballast tank, where they were homogenized and blended with pure helium. The gas mixture was then fed into a catalytic furnace filled with heated copper, where nitrogen oxides were reduced to N_2_. Lecosorb and Anhydrone reagents were used for purification from carbon dioxide and water. The gas mixture was then fed into a thermal conductivity detector to measure nitrogen content. The nitrogen content was converted into protein content (g/100 g) by multiplying the result by the appropriate coefficient (6.25 and 5.7, respectively). 

All samples obtained from malting in tested waters were mashed in an R12 mash bath manufactured by 1-CUBE using the EBC method [30]. In the mashing process, mash saccharification time was analyzed. Subsequently, the content of the mash cup was cooled, filled with distilled water to reach 450 g of mass, and paper filtered. In order to ensure high clarity, the first portions of the filtrate were recirculated. Cleared wort (200 g) was boiled in round-bottom flasks heated to boiling point in heating mantles. During the boiling process, Octavia variety hops (7% of alpha acids) at an amount of 1.6 g/L were added to the wort. Reflux condensers were used to reduce the vaporization of volatile hops compounds and boiling time was reduced to 30 min. After boiling, the wort was cooled to 20 °C and analyzed in six replicates. 

#### 2.2.7. Wort Analysis

The pH of the lab wort was measured directly with a Mettler Toledo FiveGo pH-meter. The pH meter was first calibrated with respective buffer solutions [31]. 

The lab wort color was measured by adding 0.5 g kieselguhr to reach 100 mL wort and paper filtering. The resultant wort was analyzed using a 25 mm-thick cuvette in a Beckman DU-650 UV–vis spectrophotometer [32]. 

Wort extract density was measured directly with a DMA 35 density meter [33]. 

Wort turbidity was measured with a Nefelometr Cyberscan TN 100, which applied the nephelometric method in the range 0–1000 NTU, according to EN/ISO 7027, in order to measure turbidity. 

Free mmino nitrogen (FAN) was determined by adding 2 mL diluted wort sample (1:50), 2 mL water (a blind sample), or 2 mL model glycine solution to three test tubes, as appropriate. Then, 1 mL ninhydrin was added to each test tube. Test samples were placed in a boiling water bath for 16 min. After this time, samples were cooled for 20 min in a bath at 20 °C. Subsequently, 5 mL diluting solution (2 g potassium iodate in 600 mL water and 400 mL 96% (*v/v*) ethanol) was added to each sample and mixed for 30 min. The absorbance was measured at 570 nm (Beckman DU-650 UV–vis spectrometer) against water as a reference [34]. 

Volatile compound analysis involving solid-phase microextraction gas chromatography coupled with mass spectrometry (SPME–GC–MS) 

In order to determine the volatiles, 1 g NaCl and a 2-mL sample of wort were placed in a 10-mL vial, and an internal standard solution was added (0.57 mg/L 4-methyl-2-pentanol, 0.2 mg/L anethol, and 1.48 mg/L of ethyl nonanoate; Sigma-Aldrich). An SPME device (Supelco Inc., Bellefonte, PA, USA) coated with PDMS (100 μm) fiber was first conditioned by inserting it into a GC injector port for 1 h at 250 °C. For sampling, the fiber was inserted into the headspace under stirring (300 rpm) for 30 min at 60 °C. Subsequently, the SPME device was introduced into the injector port of an Agilent Technologies 7890D chromatograph system equipped with LECO Pegasus HT, High Throughput TOFMS, and kept in the inlet for 3 min. The SPME process was automated using the GERSTEL MultiPurpose Sampler (MPS).

The tested components were separated on an Rtx-1 ms capillary column (Crossbond 100% dimethyl polysiloxane, 30 m × 0.53 mm × 0.5 μm). The detector was set at 250 °C and the column was heated using the following temperature program: 40 °C for 3 min, increment of 8 °C/min to 230 °C, then a constant temperature was maintained for 9 min. Carrier: Helium at 1 mL/min constant flow. EIMS electron energy: 70 eV; ion source temperature and connection parts: 250 °C. Analyte transfer was performed in splitless mode and the MSD was set to scanning mode (from *m*/*z* = 40 to *m*/*z* = 400). Compounds were identified using mass spectral libraries and linear retention indices, calculated from a series of n-alkanes (from C6 to C30). 

The qualitative and quantitative identification of volatile substances (nonanal, decanal, 1-nonanol, 1-decanol, 1-dodecanol, ethyl hexanoate, ethyl octanoate, ethyl decanoate, ethyl dodecanoate, ethyl tetradecanoate, isocaryophyllene, humulene, and caryophyllene oxide; Sigma-Aldrich) were based on a comparison of retention times and peak surface area, as read from sample and standard chromatograms. Other detected components were determined semi-quantitatively (μg/L) by measuring the relative peak area of each identified compound, according to the National Institute of Standards and Technology NIST database (http://webbook.nist.gov/chemistry/), in relation to that of the internal standard. Each of the test was carried out in triplicate. 

### 2.3. Statistical Analysis 

The results presented in the paper are the means of six independent repetitions determining the standard deviation. The impact of the LPGP treated water (in the air and under nitrogen) on the analyzed parameters was determined using variance analysis and the significance of differences between the means was verified by using the post-hoc Tukey’s test (Statistica 10, StatSoft Polska, Kraków). 

## 3. Results and Discussion 

### 3.1. Plasma Treated Water Analyses 

**Figure 3:**
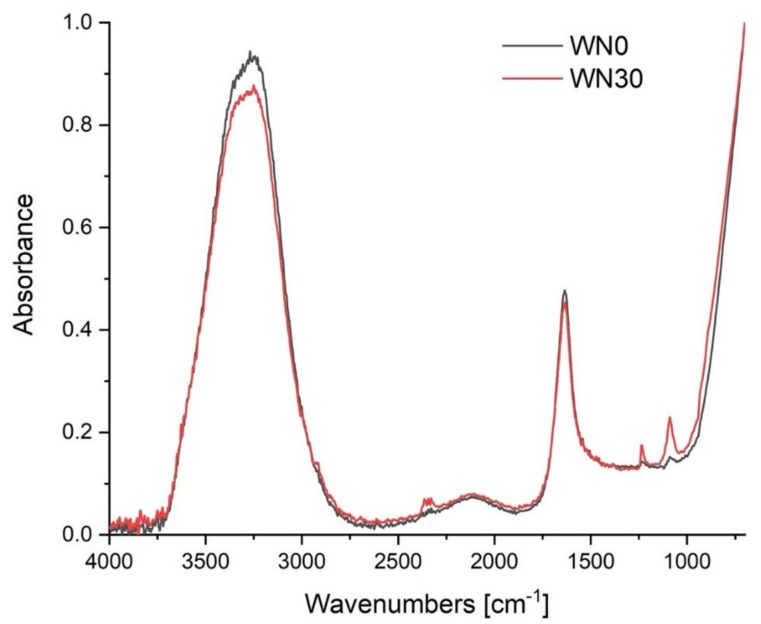
FTIR spectra of spring water from Żywiec Zdrój stored in contact with nitrogen prior to (WN0) and after treating with LPGP for 30 min (WN30).

The FTIR spectra (Figure 3) of water in the range of 4000–1250 cm^−1^ consisted of two bands of ν_OH_ combined into one broad peak and δ_OH_ band at 1633 cm^−1^. The band situated at 3243 cm^−1^ was, in fact, composed of two bands reflecting asymmetric (longer wavelength; B-band) and symmetric (shorter wavelength; A-band) vibrations in the water molecule [35]. These could be recognized after the Gaussian distribution of that band. One can see that the positions of all three bands were insensitive to treatment with LPGP and that the intensity of those bands subtly depended on it (see Table 1). 

Results for water plasma treated in the air have been published by Białopiotrowicz et al. [13]. They found that 30 min of LPGP treatment in the air favored the formation of asymmetrically vibrating water molecules with low, if any, dipole moment, making that water more hydrophobic; that is, more lipophylic (see an increase in the A/B intensity ratio). In the case of the LPGP-treated water under nitrogen, an opposite effect was observed. Plasma treatment for 30 min resulted in a decrease in the lipophylic properties of the water. 

### 3.2. Malt Analyses 

According to Kunze [2], the degree of water uptake characterizes a seed’s capacity to absorb water (i.e., its swelling ability). The speed of water absorption by a seed depends on the duration of steeping, water temperature, seed size, protein content in barley, and aeration of the seed steeped underwater and without water [36]. In the initial stages of steeping, the water absorption rate of the seed reaches approximately 1% per hour. With increasing water saturation of the seed, a subsequent increase in moisture by 1% requires more than 5 h [37]. The higher the degree of soaking the grain, the better the material for barley malt. This also indicates that barley malt is characterized by higher enzyme activity and myelin of endosperm, which is very important in the later stages of beer production [38]. Grain steeping is also significantly affected by the temperature of the steeping water. During the malting process, water at 10 to 15 °C is applied. Maintaining a constant water temperature during the entire seed steeping process is very important [39]. Before the steeping stage, the barley seed contained 11.75% moisture. Over the 5-h course of steeping, an intense increase in moisture was observed in seeds steeped in water plasma treated in the air and under nitrogen (21.5% and 22%, respectively), whereas the control sample after 5 h of steeping contained 18% moisture (see Table 2). When steeped in LPGP-treated water, after 12 h of steeping, close to 42% moisture was reached; following this, the moisture level remained unchanged until the end of steeping. In the control sample, the level of moisture at the 12th hour was close to 38%, which did not significantly increase in subsequent hours. These differences prove that the rate of the water absorption by the seed when using plasma-treated water was higher and that seed moisturizing was more efficient. This attribute is very important from the point of view of the cost-efficiency of malt production and identification of potential process shortcuts while avoiding an adverse impact on product quality. 

Water absorption by grains, one of the basic parameters analyzed in a malthouse, is closely related to their sensitivity to water. When the water sensitivity of a grain is too high, appropriate steps must be taken to limit the steeping period. The seed sensitivity to water was determined based on the impact of two volumes of water (4 and 8 mL) on the germination energy [40]. The water sensitivity of barley can be classified into low sensitivity (10%), higher sensitivity (11–25%), sensitive (26–45%), and high sensitivity (over 45%) [2]. This study proved (Table 2) that using water treated with plasma in the air and under nitrogen significantly increases the sensitivity of the grain and germination energy. 

Barley germination capacity is one of the key properties determining the quality properties of a seed in terms of the percentage of seeds producing healthy and normally growing plants [41]. This parameter is defined according to the class of barley. In classes I and II, germination capacity should not be lower than 99%, and 98% for class III [10]. Seed germination energy was determined after 3 and 5 days. High germination energy manifests the health of the seed and promises the normal progress of the malting process. In the analyzed samples, after the 3rd day of germination, a very significant difference in germination energy was observed in seeds steeped with water plasma-treated under nitrogen (95%) and in the air (97.6%). A similar effect was obtained after the 5th day of observation. A more positive impact was observed with water plasma treated in the air (99.45%) than under nitrogen (98.2%). Thus, based on the germination capacity, the barley could be categorized into classes I and II, respectively. The amount of non-germinated grain between days 3 and 5 of the process did not exceed 5%, which, according to Kunze [2], manifests the health of the seed and promises the normal progress of the malting process (Table 2). Even a slight increase in seed germination capacity achieved in the process adds substantial indicators for the malthouse to its performance and the quality of malt it produces.

The moisture content parameter is used to test the storage stability of the malt. According to Angelno [42], moisture for pale malts should range from 3.8–7.3%. In the non-treated samples, these values were slightly lower (3.6%). The moisture of the malts from the process involving water plasma-treated under nitrogen and in the air fit the standard limits (5.4% and 5.6%, respectively). Taking into account the identical conditions of malt kilning and the analysis, differences between samples might have resulted from the fact that, before starting kilning, the seeds conditioned in plasma-treated water were slightly more steeped (42%) than the control sample (39%). These differences might also be associated with the reduced availability of structurally bound water in the case of samples treated with LPGP.

The grain uniformity of brewing barley should not be lower than 90% [43]. In the tests, independently of the water applied, grain uniformity in all variants reached approximately 94%. High grain uniformity is important in the grinding process—the first stage in the malthouse—and for the adequate setting of space between rolls in a grinder. A mass of 1000 grains can be used to inform about the degree to which the seeds are filled with chemical components and their morphology, as well as finding the quantitative composition of the grinding products. In the literature, a very broad scope of results for the mass of 1000 seeds can be found. These studies had results ranging from 34.3 to 54.0 g [44,45,46]. By analyzing the mass of 1000 seeds and taking dry mass into account, significant differences were found between the control sample (51 g) and samples treated with LPGP (44 g).

One of the key parameters of malt quality is extraction yield (extractivity), which should not be lower than 79% for pale malts [2]. This parameter did not statistically significantly differ for the malts prepared within this experiment (74.1% and 74.9% for the treated samples and 72.2% for the control sample). The obtained malts had slightly lower extractivities than those described in the literature.

Protein content affects the quality of barley for beer brewing, which should range from 9.5–11.5%. If barley seed has a higher protein content, the consequent extended steeping time leads to non-uniform germination throughout the entire malting process [47]. As a result, the quality of the malt deteriorates and the beer obtained is characterized by a lower quality [48]. Barley contains 10–12% protein [49], which can be estimated by sequential extraction efficiency, according to the procedure introduced by Osborne [50]. Hordein fractions extracted in the presence of a reducing factor represent 35–55% of all seed proteins and constitute the main barley storage protein [51]. In the malting process, barley proteins are partially decomposed into amino acids and small peptides by a number of proteolytic enzymes [52]. A good quality malt contains less than half of the hordeins occurring in the original barley [53]. The average protein content of the barley used in our experiments reached 10.5% before starting the malting process. This result was within the range required by the EBC. After the malting process, the protein content of grains steeped in water treated in the air reached 9.95%, while grains steeped in water treated under nitrogen contained 10.37% protein. The results differed significantly. In the seed germination process, approximately 35–40% of proteins were transformed, and the outcome of the processes was expressed in terms of the Kolbach protein modification [54]. The level of protein in the malt was reduced against the initial content in the brewing barley seed (reaching 10.5%), as a small part of it was used for developing radicals. After the malting process, the seeds steeped in water plasma treated in the air statistically significantly differed in protein content from the seeds steeped in water plasma-treated under nitrogen (9.95% and 10.37%, respectively). During the grain germination process, about 35–40% of the proteins change, and the effect of this process is visible in terms of the degree of protein relaxation, according to Kolbach [54]. The amount of protein in malt was reduced, as compared to the initial content in the malting barley grain, due to a small part of it being used for germination [55].

### 3.3. Wort Analyses 

Ethanol, one of the main components of beer, is produced during the fermentation of the sugars developed by the hydrolysis of the starch present in wort. Starch yields mostly maltose and other products, such as dextrines, which cannot be fermented by yeast. Starch degradation proceeds in three stages: gelatinization, liquefaction, and saccharification. The time of malt saccharification in lab mashing, according to the Analytica EBC standard, cannot exceed 20 min [2,47]. Table 3 presents the results for the worts produced with the given malt variants. The wort produced in the process, after reaching 70 °C in the mash bath, was characterized by saccharification after a period shorter than 5 min. This proved the high enzymatic activity of the produced barley malt and appropriately selected malting parameters.

The results of color determination of the lab wort do not provide any specific information about the expected color of the final beer but, instead, solely indicate the type of malt used in its production. In the case of pale malts, the color of the wort should not be higher than 4 EBC units and, for malts of average color, from 5–8 EBC units [2]. In this test, the color of the produced wort did not differ significantly (values between 3–4 EBC units). All the variants could be classified as pale malts. Similarly, no statistical differences were established for wort colors after boiling with hops (1.6 g/L OKTAWIA, alpha acid content 7%; see Table 3). Prior to boiling, no statistically significant differences were reported for the samples. Turbidity is a parameter which has been analyzed in detail due to its relationship to wort quality [56]. This parameter, the type of material, the progress of the grinding process, and mashing are all very important. The turbidity of the wort is mainly influenced by the content of fats and fatty acids. The formation of a high content of proteins or protein-polyphenol complexes has been shown to contribute to increased turbidity [57].

The aromatic composition of the hopped wort depends on malt compounds, components formed during mashing, and substances derived from hops. Over 90% of the hop oil volatilizes during the boiling period and the traces are found in the hopped wort [58]. Alcohols, esters, and terpenes were the predominant aromatic compounds found in the tested wort (Table 4). No statistically significant differences were found in the content of volatile compounds in the analyzed samples; this means that the type of water used in grain soaking did not affect the aromatic composition. Moreover, no increase in the amount of oxidized compounds was noted, which would indicate the presence of a larger amount of free radicals and/or oxidizing agents in the raw materials used to obtain the wort.

Other parameters analyzed in the test, such as filtration time, the mass of the wort from the process, pH, and free amino nitrogen (FAN) content, did not show any statistically significant differences. 

## 4. Conclusions

The results of the conducted experiments indicate that water treated with low-temperature, low-pressure plasma in the air and under nitrogen can improve the malting process in the brewing industry, providing a better quality of the brewing malt. Significantly higher water uptake capacity of the grain and grain sensitivity to water, as well as energy and germination capacity for two-row spring barley grain, were observed. Quality parameters pointed to statistically significant improved moisturizing and 1000-grain mass. Other malt parameters, such as extractivity and acrospire length, did not change in any manner. Laboratory wort produced from obtained malts by the congress method did not differ statistically in terms of filtration time, pH, turbidity, color, extract, free nitrogen compounds, and aromatic composition. Thus, it has been proved that water treated with low-temperature, low-pressure glow plasma in the air and under nitrogen has no negative effect on the mashing process.

## Figures and Tables

**Table 1:** Intensity of OH stretching (bands A and B) and bending (band C) modes in the FTIR spectra of water taken in the region of 4000–1500 cm^−1^.

Sample	Band Intensity			Intensity Ratio		
	A (3494 cm^−1^)	B (3265 cm^−1^)	C (1633 cm^−1^)	A/B	A/C	B/C
WN 0	0.0810	0.3403	0.1732	0.238	0.468	1.965
WO 0 [12]				1.179		
WN 30	0.0681	0.3294	0.1693	0.207	0.402	1.946
WO 30 [12]				1.3882		

**Table 2:** The quality parameters of barley and the obtained malts.

Analyzed Parameters		Type of Water Used for Seed Soaking			Significance ^1^
		Plasma Treated Under Nitrogen	Plasma Treated in the Air	Control Sample	
Water uptake capacity of the grain [%]	5 h	21.5 a (±1.38)	21.5 a (±1.04)	18.33 b (±0.41)	**
	12 h	41.5 a (±1.38)	41.6 a (±1.63)	38.3 b (±1.03)	**
	50 h	41.5 a (±1.38)	41.6 a (±1.63)	38.75 b (±0.75)	**
Grain sensitivity to water [%]	4 mL	60.8 a (±6.27)	54.6 a (±4.72)	28.16 b (±8.13)	***
	8 mL	26.6 a (±4.13)	25.3 a (±4.08)	14 b (±1.78)	***
Energy and germination [%]	72 h	94.7 a (±0.69)	97.7 b (±0.45)	91.25 c (±0.59)	***
	120 h	98.3 a (±0.72)	99.5 b (±0.17)	97.2 c (±0.18)	***
Grading by grain size of grains [%]		93.8 a (±0.91)	94.3 a (±0.5)	94 a (±0.55)	ns
Moisture [%]		5.4 a (±0.31)	5.6 a (±0.26)	3.6 b (±1.3)	**
1000 grain weight [g]		44 a (±0.6)	44.3 a (±0.8)	51 b (±1.4)	**
Acrospire length		0.74 a (±0.04)	0.67 a (±0.04)	0.68 a (±0.1)	ns
Extractivity of malt [%]		74.1 a (±4.5)	74.89 a (±1.5)	72.25 a (±2.21)	ns
Protein content [%]		10.37 a (±0.12)	9.95 b (±0.22)	10.07 ab (±0.05)	**

^1^ Significance; **, *** indicate significance at a level of 1% and 0.5%, respectively, by the least significant difference; ns: not significant. Values with different superscript roman letters (a–c) in the same row are significantly different, according to the Duncan test (*p* < 0.05).

**Table 3:** Quality parameters of the obtained wort.

**Analyzed Parameters**	**Variety of Wort**			**Significance ^1^**
	**Plasma Treated under Nitrogen**	**Plasma Treated in the Air**	**Control Sample**	
Saccharification time (min)	>5	>5	>5	ns
Filtration time (min)	47 a (±25.63)	74 a (±33.36)	46 a (±32.62)	ns
Wort mass after filtration (g)	321.9 a (±6.98)	320.2 a (±13.8)	331.6 a (±14.1)	ns
pH	6.02 a (±0.04)	6.02 a (±0.02)	6.03 a (±0.03)	ns
Turbidity (EBC)	14.91 a (±7.15)	14.69 a (±7.46)	12.74 a (±4.6)	ns
Color (EBC units)	3 a (±0.85)	4 a (±0.58)	3 a (±0.62)	ns
Wort extract (°P)	8 a (±0.47)	8.12 a (±0.33)	8.12 a (±0.17)	ns
pH after boiling	5.95 a (±0.01)	5.99 a (±0.02)	5.98 a (±0.01)	ns
Wort color after boiling (EBC)	5 a (±0.03)	5 a (±0.02)	5 a (±0.01)	ns
Wort extract after boiling (°P)	8.5 a (±0.01)	8.5 a (±0.00)	8.5 a (±0.00)	ns
Turbidity after boiling (EBC)	139.01 a (±0.29)	137.72 a (±0.08)	177.4 b (±12.48)	**
Free amino nitrogen in wort (FAN; mg/L)	132.6 a (±19.4)	123.6 a (±19.1)	132.2 a (±8.87)	ns

^1^ Significance; ** indicates significance at a level of 1% by least significant difference; ns: not significant. Values with different superscript roman letters (a–c) in the same row are significantly different, according to the Duncan test (*p* < 0.05).

**Table 4:** Aromatic composition of the obtained hopped wort.

Compound (μg/L)	LRI^2^	Plasma Treated Under Nitrogen	Plasma Treated in the Air	Control Sample	Significance ^1^
**Esters**					
Ethyl octanoate	1180	3.35 a	0.00 b	2.68 a	**
Ethyl 2-methyloctanoate^3^	1209	8.4	7.8	6.1	ns
Propyl nonanoate^3^	1373	0.09	0.08	tr.	ns
Ethyl decanoate	1397	0.08	tr.	tr.	ns
Ethyl dodecanoate	1581	0.07	tr.	tr.	ns
Ethyl tetradecanoate	1790	tr.	tr.	tr.	ns
**Alcohols**					
1-Hexanol, 2-ethyl-^3^	1020	129.6	101.2	133.3	ns
1-Octanol, 2-methyl-^3^	1119	27.6	21.6	25.6	ns
1-Nonanol	1156	143.2	95.5	134.1	ns
1-Heptanol, 2-propyl-^3^	1203	0.08	0.12	0.18	ns
Ethanol, 2-[(2-ethylhexyl)oxy]-^3^	1226	1.38	3.18	2.40	ns
1-Decanol	1272	0.59	1.28	1.34	ns
1-Undecanol^3^	1368	0.10	0.12	0.55	ns
2-Dodecanol^3^	1417	tr.	0.06	0.06	ns
1-Dodecanol	1480	tr.	tr.	0.07	ns
**Terpenes**					
Isocaryophyllene	1414	tr.	tr.	tr.	ns
trans-α-Bergamotene^3^	1432	tr.	tr.	tr.	ns
Humulene	1455	0.59a	0.47a	tr. b	*
D-Germacrene^3^	1485	tr.	tr.	tr.	ns
α-Farnesene^3^	1490	0.59	1.66	2.20	ns
Caryophyllene oxide	1578	0.05	0.07	tr.	ns
Humulene epoxide II^3^	1606	0.18	0.15	tr.	ns
**Carbonyl Compounds**					
Nonanal	1102	8.37	3.82	5.31	ns
Decanal	1182	24.8	28.2	25.4	ns
9-Oxabicyclo [6.1.0]nonan-4-one^3^	1212	0.30	0.86	0.65	ns
**Other Compounds**					
2-Acetyl-2,3,5,6-tetrahydro-1,4-thiazine^3^	1345	tr.	tr.	tr.	ns
Octane, 1,1′-oxybis-^3.^	1657	tr.	tr.	tr.	ns

^1^ Significance; *, ** indicate significance at levels of 5% and 1%, respectively, by least significant difference; ns: not significant. Values with different superscript roman letters (a–c) in the same row are significantly different, according to the Duncan test (*p* < 0.05). ^2^ LRI, linear retention index. ^3^ Determined semi-quantitatively by measuring the relative peak area of each identified compound, according to the NIST database, in relation to that of the internal standard. tr, trace amounts.
